# PD126 A Systematic Review Of Interval Cancer Rates In Colonoscopy Screening For Colorectal Cancer

**DOI:** 10.1017/S0266462324003635

**Published:** 2025-01-07

**Authors:** Chisato Hamashima, Koichiro Abe, Teruhiko Terasawa, Satoyo Hosono, Takakafumi Katayama, Keika Hoshi, Toshihiro Tadano, Seiju Sasaki

## Abstract

**Introduction:**

Colonoscopy screening has been suggested as a potential primary screening method for colorectal cancer (CRC). A 10-year screening interval has been recommended by some academic societies, but the scientific evidence for this needs to be more comprehensive. We performed a systematic review of interval cancer rates in colonoscopy screening in an asymptomatic population.

**Methods:**

We searched the PubMed and Ichushi-Web bibliographic databases for papers published from inception to September 2022. The search terms were “colorectal cancer screening”, “interval cancer”, and “total colonoscopy”. Randomized controlled trials and observational studies were included. The target population was an asymptomatic average-risk population with no polyps or adenomas at the index colonoscopy. Advanced neoplasia (AN) was defined as CRC or an adenoma at least 10 mm in size with a villous component or high-grade dysplasia. The incidence rates of interval CRC and AN per 100,000 person-years (PYs) were estimated.

**Results:**

Of the 694 potentially eligible articles, 15 were included. Rates of interval CRC and AN were reported in 15 and 11 studies, respectively. For the meta-analysis, 287,602 patients with negative colonoscopy results were included, with an average follow-up of 7.98 years. Negative colonoscopy results were defined as no adenoma present. The incidence rate per 100,000 PYs was 9.57 (95% confidence interval [CI]: 2.06, 29.94) for interval CRC and 311.5 (95%CI: 153.4, 550.7) for AN. Similar results were obtained even when a negative colonoscopy result was defined as no polyps present.

**Conclusions:**

The selected studies were heterogeneous with respect to the target population, follow-up years, and patient characteristics. Although interval AN was reported in all studies, the interval cancer rate was low after a negative result at the index colonoscopy. While the screening interval might be defined as long term, a definitive screening interval could not be recommended because of insufficient evidence.

